# New York

**Published:** 1895-11

**Authors:** Jos. N. B. Rawle


### LEGISLATION.

### NEW YORK.

A VETERINARY surgeon by the grace of the Legislature of the State of New York becomes a "veterinarian," and the authority that creates written laws thought wise and disembogued the common appellation, veterinary surgeon, and created the new and highly distinctive doctorate, veterinary physician and surgeon, *fieri non debet, sed factum valet.*

Statutory enactments of New York are not extra-territorial, consequently of no value to the non-resident, where custom would have a like effect to the legal act here, so the establishment of a law in New York comes with no greater force than a compliment to the graduate and non-graduate "veterinary physician and surgeon" who "is duly registered" and "legally qualified" (Laws of New York, 1895).

Strange as it may appear that a man must be "duly registered" before practising carries with it by the same power a legally qualified authority, but would not the profession at large wonder if on any of the examining boards in this country it appeared that one of the examiners was not "duly registered," and, therefore, not "legally qualified?" but such a thing may happen.

Does a veterinary physician and surgeon rise to a higher degree than before such enactments, criminal and quasi criminal in their nature, establishing a certain standard of knowledge equivalent to admit individuals to practice will never affect or accomplish the great and intended result, advancement, progressiveness?

What a veterinarian does is of more practical moment than what he knows. College degrees and diplomas should be uniform. The courses of one should be the courses of all, authority of one State should be the authority of all; and then the trained men would outstep the quack and ignorant, who foister themselves upon the community either under the guise of a diploma or the certificate of an examining board or by favor of being "duly registered."

The national department of all institutes of science should be under the supervision of a department created at Washington, D. C.; all examinations to emanate therefrom after passing the examination required by the Board of Regents of the State wherein the institution of learning is located, and would act as the qualification to practise in any State of the Union.

After the passage of efficient laws and the increase in preliminary education than now generally allowed, and offering facilities to those who cannot attend full courses at colleges to obtain so much of the instruction there as they can and allow them to appear before a State body to be examined, and after such examination to be subjected to another by the national examining board, and in that way the profession would obtain true and worthy men, and would reap untold benefits.

Some of our colleges have graduated men who, under the provisions of law and the assistance of the term-fees, answering the roll-call, and marking the soft side of the board seat for required courses, become "legally qualified" to go forth and do havoc among the animal world. One case which illustrates how forcible this truth appears. In Broom *vs.* Reed, 69 Hun, 426, the Court held "a veterinary surgeon in order to recover for the value of his services must possess and exercise a reasonable degree of learning and skill, and use reasonable and ordinary care and diligence in its application and exercise." There must be a wholesome discrimination in the selection of students if we follow this dictum, only those to be taken who possess a reasonable degree of learning. The University of the State of New York has set the pace, and there will be a decrease in the number of matriculants in New York colleges; the cause, a lack of preliminary education. Better few and capable than large and ignorant classes.

Want of care, diligence, and skill was held in a case where a veterinarian left the animal treated in a dangerous condition and failed to resume treatment, and where he had prescribed for a disease different from that from which the animal suffered.

How often has it occurred in the practice of all when we come in contact with some of the "legally qualified" just such a case.

Raise the standard among your societies; stop bickering; do not abuse one another before the public; speak ill of no one; make the people respect you as a man of science; let others speak of your greatness and eventually you will become great. And be the true veterinarian in spirit and deed.

Jos. N. B. Rawle, L.B., M.D.S., V.S.

